# Effect of Casirivimab/Imdevimab Treatment on Serum Type I Interferon Levels in SARS-CoV-2 Infection

**DOI:** 10.3390/v14071399

**Published:** 2022-06-27

**Authors:** Kentaro Nagaoka, Hitoshi Kawasuji, Yusuke Takegoshi, Yushi Murai, Makito Kaneda, Akitoshi Ueno, Yuki Miyajima, Hideki Niimi, Yoshitomo Morinaga, Yoshihiro Yamamoto

**Affiliations:** 1 Department of Clinical Infectious Diseases, Graduate School of Medicine and Pharmaceutical Sciences, University of Toyama, Toyama 930-0194, Japan; kawasuji@med.u-toyama.ac.jp (H.K.); takegos@med.u-toyama.ac.jp (Y.T.); yushimurai@gmail.com (Y.M.); kane2214@med.u-toyama.ac.jp (M.K.); aueno@med.u-toyama.ac.jp (A.U.); myuki@med.u-toyama.ac.jp (Y.M.); yamamoto@med.u-toyama.ac.jp (Y.Y.); 2 Department of Clinical Laboratory and Molecular Pathology, Graduate School of Medicine and Pharmaceutical Sciences, University of Toyama, Toyama 930-0194, Japan; hiniimi@med.u-toyama.ac.jp; 3 Department of Microbiology, Graduate School of Medicine and Pharmaceutical Sciences, University of Toyama, Toyama 930-0194, Japan; morinaga@med.u-toyama.ac.jp

**Keywords:** COVID-19, type I interferon, casirivimab, imdevimab, SARS-CoV-2, antibody-dependent enhancement

## Abstract

The effects of casirivimab and imdevimab (C/I) on the innate immune response against SARS-CoV-2 infection remain unclear. We evaluated the effect of C/I on type I interferon (IFN-I) and cytokines in patients with SARS-CoV-2 infection. This prospective observational study recruited consecutive patients hospitalized with SARS-CoV-2 infection. Blood levels of IFN-I and cytokines before and after C/I administration were assessed using enzyme-linked immunoassay. The study enrolled 29 patients in the C/I group. In addition, 11 patients who received remdesivir and dexamethasone (R/D group) during the early phase (≤5 days after the onset of symptoms) were included as a comparator group. After treatment, IFN-α and IFN-β levels decreased significantly in both the C/I group and R/D group, whilst the post-treatment neutrophil-to-lymphoid ratio increased in the early C/I group but not the R/D group. In the C/I group, temporal temperature elevation and hypoxemia were observed after treatment in 58.6% and 41.4% of the cohort, respectively. However, most patients recovered by 5 days after treatment. This study could demonstrate the high therapeutic effect of C/I with an antibody-dependent enhancement-like response and decreased IFN-I production, which was likely due to the immediate induction of an antibody-dependent immune response against SARS-CoV-2.

## 1. Introduction

Severe acute respiratory syndrome coronavirus 2 (SARS-CoV-2) causes coronavirus disease 2019 (COVID-19), which has spread worldwide, increased the national disease burden, and continues to pose a serious global public health threat. Amid the current COVID-19 pandemic, SARS-CoV-2 monoclonal antibodies (mAb) infusions were developed as a novel class of antiviral drugs [1,2]. Neutralizing mAbs are recombinant proteins derived from the B cells of convalescent patients or humanized mice and are known to promote passive immunity [3]. The main mechanisms of passive immunity of mAbs include the following: (1) antibody-mediated neutralization against the virion, which can prevent target cell binding and/or fusion; (2) antibody-mediated opsonization against the virions or infected cells for phagocytic uptake; (3) antibody-dependent cellular phagocytosis; (4) complement-dependent cytotoxicity; and (5) antibody-dependent cellular cytotoxicity. Accompanied by these effects, viral pathogenesis could be facilitated via increased viral uptake and replication, or via increased immune activation (cytokine production, immune cell activation, complement cascade activation), collectively termed “antibody-dependent enhancement (ADE)” [3].

The combination of casirivimab and imdevimab (C/I) acts as a mAb against COVID-19, consisting of two recombinant human IgG1 monoclonal antibodies that bind non-competitively to non-overlapping epitopes of the spike protein receptor-binding domain of SARS-CoV-2 [4]. Several studies have reported the high therapeutic efficacy of C/I, and a low incidence of ADE, resulting in decreased hospitalization and mortality rates in out-patients with mild to moderate COVID-19, particularly in high-risk patients [5,6,7,8,9,10,11]. However, the effect of C/I on immune activity in real-world patients with COVID-19 remains largely unknown.

This study assessed the effect of C/I on the early immune response against SARS-CoV-2 infection, including type I interferon (IFN-I) and pro-inflammatory cytokines. IFN-I, which mainly consists of IFN-α and IFN-β, has emerged as a crucial contributor to the initial immune response against SARS-CoV-2 infection [12,13,14]. IL-6 and CXCL10 are pro-inflammatory cytokines that act as initial immune triggers of the cytokine storm in SARS-CoV-2 infection [15]. Because these biomarkers are potential treatment targets in SARS-CoV-2 infection [16,17,18,19,20], we considered it important to assess the association between C/I and these biomarkers.

## 2. Materials and Methods

### 2.1. Study Design

This study was conducted as part of the Toyama University COVID-19 cohort study, an investigator-initiated, prospective, single-center study primarily designed to investigate the clinical, epidemiological, radiological, and microbiological features of COVID-19. In this study, patients were diagnosed with COVID-19 based on quantitative reverse transcription polymerase chain reaction (RT-qPCR) results. Serum samples were collected and stored at −80 °C after each laboratory examination. This study was approved by the Ethical Review Board of the University of Toyama (R2019167), and written informed consent was obtained from all patients.

The inclusion criteria were: (1) men or women aged 18 years or older; (2) hospitalized at Toyama University Hospital, Toyama, Japan, between July 2021 and October 2021 (during the endemic period in Toyama, which was caused by the SARS-CoV-2 B.1.617.2 lineage); (3) positive diagnosis of SARS-CoV-2 infection via qPCR using nasal swab specimens; and (4) blood samples collected after admission to our hospital. Patients who had received a vaccine or any treatment against SARS-CoV-2 before admission, or those who participated in another clinical trial, were excluded.

### 2.2. Study Participants and Protocol

Patients were assigned in a non-randomized manner to either the C/I group, which received C/I (600 mg each/100 mL saline), or the (R/D group, which received remdesivir (200 mg on day 1 and 100 mg/day from days 2–5) and dexamethasone (6.6 mg/day from days 1–5), based on their choice of therapy. The length of treatment period for RDV/DMX was recommended as 5 days in Japan at the time. In addition, subjects who improved without receiving any treatment were assigned to the untreated group. Patients treated with C/I and remdesivir were excluded from the study (*n* = 2). Furthermore, the C/I group was divided into two groups according to the timing of C/I administration, i.e., the early phase or the late phase. Although the cut-off for mAb eligibility of 7–10 days after the onset of symptoms (i.e., on clinical day 7–10) has been established worldwide, earlier administration of C/I has been reported with favorable outcomes [8,11]. During the study period, we treated the patients with hypoxemia with R/D and those without hypoxemia with C/I. In our study, hypoxemia often occurred in the later phase of SARS-CoV-2 infection (after clinical day 6). A significant subset of the C/I group (*n* = 23, 77%; 23) was treated with C/I on clinical day 5, while 33% of R/D group was treated during the same period. In the R/D group treated after clinical day 6, baseline evaluation was unavailable for nine patients (47.4%) because they had received R/D prior to admission to our hospital. Based on these, we defined “clinical day 5” as a cutoff for the timing of C/I treatment, “clinical day ≤ 5” as the early phase, and “clinical day ≥ 6” as the late phase.

Data on patient demographics, comorbidities, clinical presentation, laboratory findings, therapy regimen, and prognosis were collected from medical charts. Deterioration of fever after treatment was counted when body temperature elevated higher (≥0.1 °C) than the peak temperature before treatment. Hypoxemia requiring oxygen therapy was defined as an SpO2 of ≤93% at rest/motion in room air. This is a universally accepted criterion for the initiation of oxygen therapy in patients with COVID-19 [21].

### 2.3. Blood Samples

A 1.0 mL serum sample was collected from each patient and divided into three tubes, one of which was used for cytokine measurements (see below).

### 2.4. Cytokine Measurement and Protocol

Serum cytokines and chemokines (IFN-α, IFN-β, IL-6, CXCL10, and VEGF) were measured using commercially available ELISA assays, as per the manufacturer’s instructions. The levels of IFN-α, IFN-β, IL-6, CXCL10, and VEGF were measured using the VeriKine-HS Human IFN Alpha All Subtype ELISA Kit (PBL Assay Science, Piscataway, NJ, USA), VeriKine-HS Human IFN Beta Serum ELISA Kit (PBL Assay Science), AuthentiKine™ Human IL-6 ELISA Kit (Proteintech, Rosemont, IL, USA), Human CXCL10/IP-10 ELISA Kit (Proteintech), and AuthentiKine™ Human VEGF ELISA Kit (Proteintech), respectively. Each sample was measured during the first thaw cycle. If an analyte signal was below the background signal, it was set to zero, and if the signal was detectable but below the manufacturer’s lower limit of quantification, it was set to the lower limit of detection.

To assess the effect of C/I on IFN-I and cytokine levels, we measured IFN-I and cytokine levels before and after treatment. For assessment of those values before treatment, we used serum samples which were collected within 24 h of C/I administration. For the assessment after treatment, we used serum samples which were collected 3 days after C/I administration. To assess the effect of R/D on IFN-I and cytokine levels, we measured each value of IFN-I/cytokines before and after the treatment, within 24 h before RDV administration, and 5 days after initiation of the therapy. To assess each value of IFN-I/cytokine in the untreated group, serum collected before clinical day 5 was used. The follow-up evaluation was unavailable for nine patients (52.9%) of untreated patients because they had discharged a few days after initial assessment. Thus, we measured each value of IFN-I/cytokines at single point with the untreated patients.

### 2.5. Quantitative Reverse Transcription Polymerase Chain Reaction

RT-qPCR to detect SARS-CoV-2 was performed as previously described [22]. Briefly, RNA was extracted from 140 μL of blood serum or supernatant of nasal swab specimens using the QIAamp ViralRNA Mini Kit (QIAGEN, Hilden, Germany) or Nippongene Isospin RNA Virus (Nippongene, Tokyo, Japan), respectively, according to the manufacturer’s instructions. The viral loads of SARS-CoV-2 were quantified via RT-qPCR using an N2-gene-specific primer/probe set according to the Japan National Institute of Infectious Diseases protocol [23]. AcroMetrix COVID-19 RNA Control (Thermo Fisher Scientific, Carlsbad, CA, USA) was used as the positive control. The detection limit was approximately 0.4 copies/μL (2 copies/5 μL).

### 2.6. Statistical Analysis

Background factors were expressed as medians (interquartile range) or numbers (percentages). The difference between the two groups was tested using the Wilcoxon rank-sum test or Fisher’s exact test. The Mann–Whitney U test with Bonferroni correction was used to compare nominal variables among the 3 groups. Statistical significance was set at *p* < 0.05. The JMP Pro 16 statistical program (SAS Institute Inc., Cary, NC, USA) and GraphPad Prism (version 9.0; GraphPad Software, San Diego, CA, USA) were used for statistical analyses.

## 3. Results

### 3.1. Study Participants

**Figure 1:**
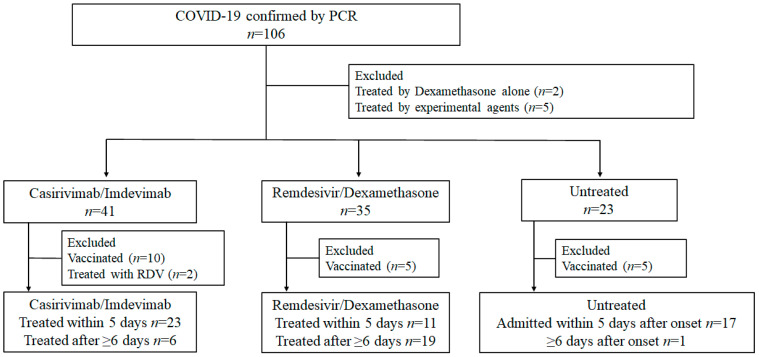
Flow chart illustrating distribution of this study.

Detailed information on enrollment is presented in Figure 1. In the C/I group, 23 patients received C/I during the early phase (early C/I group), and six patients received C/I during the late phase (late C/I group). The following patients were also considered as a comparator group for the early C/I group: a) 11 patients treated with RDV/DMX in the early phase, and b) 17 patients in the untreated group who were admitted to our hospital before clinical day 5.

### 3.2. The Effect of Casirivimab and Imdevimab on the Immune Response in the Early Phase of SARS-CoV-2 Infection

The clinico-microbiological characteristics, treatment details, and clinical outcomes of the early C/I, R/D, and untreated groups are summarized in Table 1. Age, sex, medical comorbidities, and initial nasopharyngeal viral load were similar between the three groups. Body mass index was significantly higher in the R/D group compared to the other two groups. In the early C/I group (*n* = 23), deterioration of fever after C/I administration was observed in 14 patients (60.9%), whereas fever degraded in all the patients (*n* = 11) after the initiation of RDV/DMV in the R/D group (100%). Moreover, eight (34.8%) patients in the early C/I group (*n* = 23) developed hypoxemia after C/I administration. However, these clinical symptoms had improved within 2–3 days, which resulted in a shorter hospital stay compared with the early R/D group; the total hospitalization duration was 5 days (5–7 days) in the early C/I group and 11 days (11–15 days) in the R/D group (*p* < 0.001). The total duration of oxygen therapy was significantly shorter in patients with hypoxemia in the early C/I group [2.0 (1.8–2.3)] compared to the early R/D group [9 (4.8–12.3)] (*p* < 0.001).

**Figure 2:**
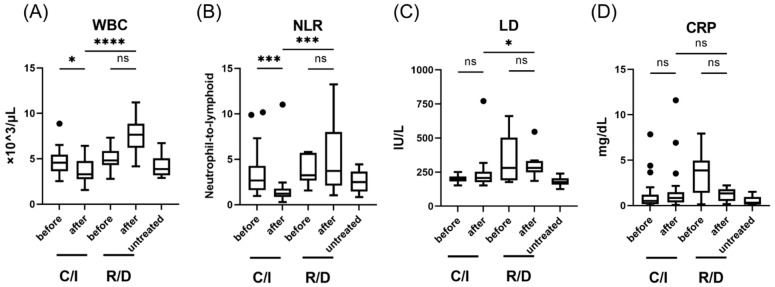
Laboratory findings in patients with SARS-CoV-2 infection, treated in the early phase (within 5 days after the onset of symptom); WBC (**A**), NLR (**B**), LD levels (**C**), and CRP levels (**D**). Each level was evaluated at the time point before and after the treatment; within 24 h before the initiation of therapy, 3 days after C/I administration or 5 days after R/D initiation. Data are presented as Tukey boxplots and individual values. Non-parametric Mann–Whitney test with Bonferroni correction was used to compare values between groups. *, *p* < 0.05. ***, *p* < 0.001. ****, *p* < 0.0001. C/I, casirivimab/imdevimab group; NLR, neutrophil-to-lymphoid ratio; ns, not significant; R/D, remdesivir/dexamethasone group; WBC, white blood cell count.

**Figure 3:**
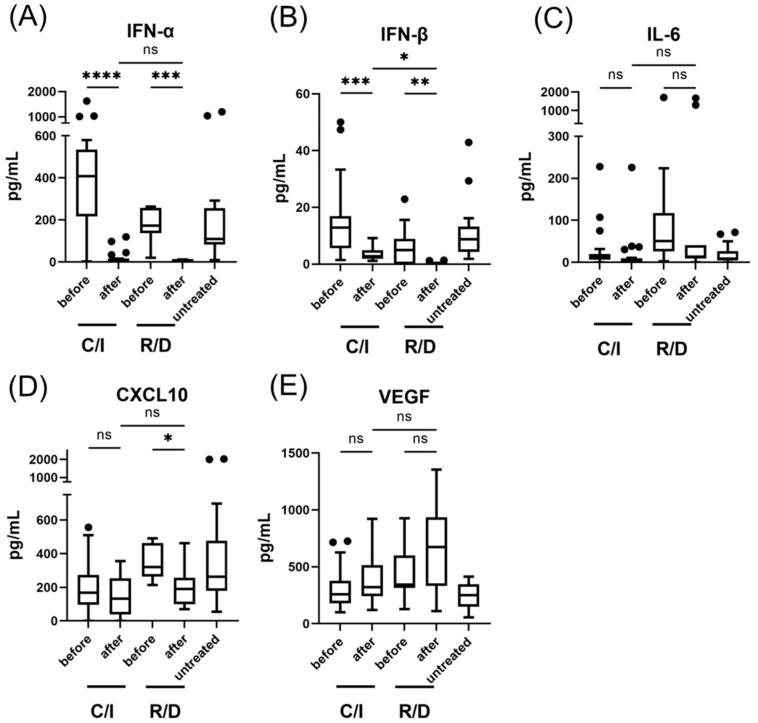
Serum type I IFN and pro-inflammatory cytokine levels in patients with SARS-CoV-2 infection, treated in the early phase (within 5 days after the onset of symptom); IFN-α levels (**A**), IFN-β levels (**B**), IL-6 levels (**C**), CXCL10 levels (**D**), and VEGF levels (**E**). Each level was evaluated at the time point before and after the treatment; within 24 h before the initiation of therapy, 3 days after C/I administration or 5 days after R/D initiation. Data are presented as Tukey boxplots and individual values. Nonparametric Mann–Whitney test with Bonferroni correction was used to compare values between groups. *, *p* < 0.05. **, *p* < 0.005. ***, *p* < 0.001. ****, *p* < 0.0001. C/I, casirivimab/imdevimab group; IFN, interferon; ns, not significant; R/D, remdesivir/dexamethasone group.

The white blood cell (WBC) count and neutrophil-to-lymphocyte ratio (NLR) decreased significantly after treatment in the early C/S group (from 4.57 (3.76–5.37) × 10^3^/μL to 3.28 (2.79–4.56), *p* <0.05; from 2.67 (1.62–4.03) to 1.20 (0.87–1.76), *p* < 0.001) (Figure 2). After treatment, the WBC, NLR, and LD were significantly lower in the early C/S group than in the early R/D group. Figure 3 shows the effect of C/I compared to R/D on IFN-I and pro-inflammatory cytokines during the early phase of SARS-CoV-2 infection compared to untreated patients. A significant decrease in IFN-α and IFN-β was observed after treatment in the early C/I group (from 408 (225–531) pg/mL to 6.45 (4.58–12.75) pg/mL, *p* < 0.0001; from 12.8 (5.76–15.8) to 2.80 (2.03–4.70), *p* < 0.001), as well as in the early R/D group (from 173 (142–251) pg/mL to 2.0 (1.03–4.95) pg/mL, *p* < 0.001; from 4.92 (0.71–8.12) to 0 (0–0), *p* < 0.01). CXCL10 was significantly decreased after treatment only in the early R/D group (from 320 (271–404) pg/mL to 190 (127–251) pg/mL, *p* < 0.05). After treatment, the serum levels of IFN-β were significantly higher in the early C/S group than in the R/D group (2.8 (2.03–4.70) pg/mL vs. 0 (0–0) pg/mL, *p* < 0.05).

### 3.3. The Effect of C/I in the Late Phase on the Immune Response against SARS-CoV-2 Infection

**Figure 4:**
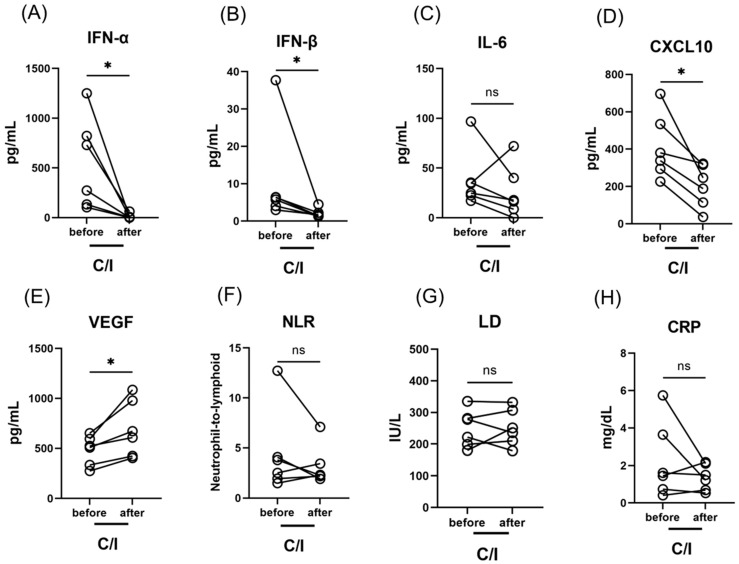
Serum type I IFN, pro-inflammatory cytokine levels and laboratory findings in patients with SARS-CoV-2 infection, treated by casirivimab/imdevimab in the late phase (6–10 days after the onset of symptom); IFN-α levels (**A**), IFN-β levels (**B**), IL-6 levels (**C**), CXCL10 levels (**D**), VEGF levels (**E**), NLR (**F**), LD levels (**G**), and CRP levels (**H**). Each level was evaluated at the time point before and after the treatment; within 24 h before the initiation of therapy, and 3 days after C/I administration. Data are presented as Tukey boxplots and individual values. The Wilcoxon rank sum test was used to compare values between groups. *, *p* < 0.05. C/I, casirivimab/imdevimab group; IFN, interferon; NLR, neutrophil-to-lymphoid ratio; ns, not significant; WBC, white blood cell counts.

The clinical characteristics and outcomes of patients treated with C/I during the late phase (late C/I group) are summarized in Table 2. Notably, fever temporarily elevated after the administration of C/I in three of the six patients in this subgroup. Moreover, four patients developed hypoxemic respiratory failure after treatment and required oxygen therapy for 2–4 days. However, all patients in the late C/I group improved and were discharged within six days of treatment. In the late C/I group, IFN-α, IFN-β and CXCL10 levels decreased significantly after treatment (from 502 (168–799) pg/mL to 2.92 (0.08–6.2) pg/mL, *p* < 0.05; from 6.05 (4.49–6.40) pg/mL to 1.56 (1.34–2.08), *p* < 0.05; from 359 (305–496) pg/mL to 217 (133–299) pg/mL, *p* < 0.05), while VEGF increased (from 516 (379–577) pg/mL to 640 (470–902) pg/mL, *p* < 0.001) (Figure 4).

## 4. Discussion

During the fifth wave of the COVID-19 pandemic (July 2021 to October 2021) in Toyama, Japan, high-risk patients often presented with SARS-CoV-2 infection due to the B. 1. 617. 2 (delta) variant. Hospitalization was mandatory for all patients with SARS-CoV-2 infection during the study period, even in mild cases. Therefore, we could not assess the effect of C/I on the reduction in hospitalization rate. Instead, we closely monitored the condition of patients during their hospital stay. In our study cohort, temporal exacerbation of fever and respiratory conditions after treatment was frequently observed in the C/I group (58.6% and 41.4%, respectively), which was similar to the incidence rates reported in other studies [24,25]. Yoshida et al. [24] reported that 49.0% of the patients treated with C/I developed body temperature elevation (≥1.0 °C) after C/I administration, and 21.9% of those developed temporal hypoxemia. However, 99.0% of patients recovered within 4 days of treatment. Similarly, Cicchitto et al. [25] reported that one of four patients with SARS-CoV-2 infection due to the delta variant developed hypoxemia after C/I administration. However, the incidence of these ADE-like responses after C/I administration is not frequent in patients infected with other SARS-CoV-2 variants [7,8,9,10,11]. Based on these findings, the ADE-like response observed in our study may be due to the strain-dependent virulence of the delta variant [26]. Considering the favorable outcomes observed in the C/I group, we suggest that the temporal exacerbation in body temperature and respiratory condition after treatment could be categorized as “ADE-like response” rather than “ADE response”, since “ADE” implies negative adverse effect.

We demonstrated that serum IFN-I levels significantly decreased after C/I treatment in patients with mild-to-moderate SARS-CoV-2 infection in both the early phase and the late phase. Although ADE-like responses were frequently observed, all patients in the C/I group improved within 2–4 days of treatment, which supports that the decrease in IFN-I levels might be a part of the favorable recovery process of SARS-CoV-2 infection. For this explanation, we consider that two mechanisms might be important, i.e., (1) antibody-mediated neutralization of the virion, and (2) antibody-dependent immunity responsivity.

It is widely known that the disease severity of SARS-CoV-2 infection is strongly associated with a dysregulated innate immune response, including an increased production of IFN-I and pro-inflammatory cytokines [27]. In innate immunity against SARS-CoV-2 infection, IFN-I plays a critical role, since it inhibits viral replication in infected cells and has a defensive role in uninfected cells [12,13,14]. Impairment of the IFN-I response relative to the onset of symptoms probably results in high viral replication and produces an exaggerated inflammatory response [28]. The infected cell types that produce IFN-I are different. IFN-α is mainly produced by circulating hematopoietic cells, whereas IFN-β is produced by a broad range of cell types [29]. Previously, we reported that serum IFN-α levels were elevated in patients in the early phase (clinical day ≤ 5) of mild to moderate SARS-CoV-2 infection, which is highly associated with the development of hypoxemic respiratory failure and viremia [30]. Considering the distribution of drug delivery, C/I might strongly neutralize the virion in the blood compared to those in the nasopharynx or other organs. These may contribute to the reduction of serum IFN-I levels via direct antibody-mediated reduction of the viral load in the blood. The decrease in IFN-α was likely to be more significant than that in IFN-β, which may be derived from the infected cell types that produce IFN-I.

In addition to the direct antibody-mediated reduction of viral load, we suggest that neutralization by C/S via induction of an antigen-dependent immune response is an important possible mechanism for the decrease in IFN-I found here. NLR elevation and ADE-like responses after treatment in the C/I group were likely derived from an antigen-dependent immune response. Robust immunity against SARS-CoV-2 is an adaptive (passive) immune response that is induced by variable antibodies generated shortly after the onset of infection [31,32]. We had previously reported that the time-course of neutralization via adaptive immune response against SARS-CoV-2 varied by disease severity [33]. An elevation in antiviral activity in serum was delayed in patients with hypoxemic respiratory failure due to SARS-CoV-2 infection compared to those in mild cases [33]. Based on these findings, we postulate that the effect of C/I on NLR, IFN-I, and ADE-like response might reflect the immediate activation of antigen-dependent immunity by C/I, which induces a significant shift in the adaptive immune response against SARS-CoV-2, thus reducing the innate immune response, including decreasing IFN-I production.

In this study, we also examined the levels of IL-6, CXCL10, and VEGF to assess the effect of C/I on immune activity related to cytokine storms and vessel invasion. After C/I administration in the late phase, we found significant decrease with CXCL10 levels and significant elevation of VEGF levels (Figure 4). These might indicate that C/I administration also potentiate to affect the production of these cytokines. However, the interaction between these biomarkers and C/I treatment in the early phase was unclear, possibly due to the relatively small sample sizes per subgroup. Further investigation is necessary to examine the detailed mechanism of antigen-dependent immunity induced by C/I.

To our knowledge, this is the first study to demonstrate the therapeutic effect of C/I on the innate immune response. Together with close monitoring of vital signs, we propose that C/I immediately induces an antibody-dependent immune response against SARS-CoV-2, with an ADE-like response and reduction of innate immune pathways, including changes in IFN-I production. Our study revealed several points that are important for understanding the interaction between C/I and other treatment targets. In a descriptive review of the literature on the role of IFN-I in treating COVID-19, a positive outcome of IFN-α and IFN-β, administered via inhalation or intramuscularly in the early phase of COVID-19, was observed [16,17,18,19]. IL-6 is one of the major therapeutic targets for SARS-CoV-2 infection, which can be treated with tocilizumab [20]. The results of our study indicate that combination therapy of IFN-I and C/I might be less effective, and tocilizumab or other immunosuppressants might reduce the ADE-like response induced by C/I. Since the indication for SARS-CoV-2 mAbs has been in discussion whether to extend to severe-fatal cases with standard care (RDV, DMX, and other immunosuppressants) [34], the data in our study might contribute to the development of future combination therapies with mAbs and other anti-viral agents or immunosuppressants. Although the application of C/I was limited to the SARS-CoV-2 variants other than omicron variant, we believe that our findings might still be contributive for understanding the part of mechanisms of mAb treatment against SARS-CoV-2.

The present study had several limitations. First, the single-center observational study design with a relatively small sample size may have resulted in a selection bias. Second, we validated IFN-I and cytokine levels by using serum samples, that were not strictly stored immediately after drawing (immediately stored in a 4 °C freezer, and then transferred to a −80 °C freezer). Third, we could only assess the untreated group at a single time point, according to the short length of hospital stay (2–7 days). However, this study could include various unvaccinated patients in the same endemic period, which would minimize bias due to vaccine- or strain-dependent SARS-CoV-2 virulence. Further, because the methodology measuring each IFN/cytokine had been confirmed as reported in the previous study [30], we believe that these limitations would not have significantly affected our outcomes.

## 5. Conclusions

In conclusion, this study demonstrated the high therapeutic effect of C/I with an ADE-like response and reduction of IFN-I production, which was likely due to the immediate induction of an antibody-dependent immune response against SARS-CoV-2. These findings will encourage further research on the effects of mAbs on innate and adaptive immune responses, which is crucial for the establishment of a novel therapeutic approach.

## Figures and Tables

**Table 1:** Clinical feature of patients with SARS-CoV-2 infection in the early phase.

	Total (*n* = 51)	C/I (*n* = 23)	R/D (*n* = 11)	Untreated (*n* = 17)
Age, years	51 (40–55)	53 (50–56)	52 (43–57)	38 (30–50)
Sex; male/female	32/19	11/12 **	10/1	11/6
Underlying disease				
None	29 (57)	15 (65)	5 (45)	9 (53)
Hypertension	9 (18)	4 (17)	4 (36)	1 (6)
Diabetes mellitus	2 (4)	0 (0)	1 (9)	1 (6)
Body mass index (kg/m^2^)	21.7 (20.0–25.0)	21.7 (19.1–24.8)	27.0 (24.8–30.9)	20.8 (19.3–21.5)
Initial nasopharyngeal-viral load (log copies/μL)	5.0 (4.3–5.7)	5.2 (4.7–5.8)	4.7 (4.0–5.3)	5.0 (4.5–5.6)
Deterioration of fever after treatment ^a^	-	14 (61) **	0 (0)	-
<1.0 °C	-	7 (30)	0 (0)	-
≥1.0 °C	-	7 (30)	0 (0)	-
Oxygen therapy				
required oxygen therapy	18 (35)	8 (35) **	10 (91)	-
duration period of oxygen therapy (days)	3 (2–10)	2 (1.8–2.3) **	9 (4.8–12.3)	-
invasive positive pressure ventilation	2/2	0	2/2	-
The length of hospital stay (days)	6 (5–8)	5 (5–7) **	11 (11–15)	4 (4–7) **
Death within 30-days after onset	0 (0)	0 (0)	0 (0)	0 (0)

Continuous variables are reported as median [interquartile range (IQR) 25–75]. Categorical variables are reported as number (percentages). ^a^, deterioration of fever was defined as at least ≥0.1 °C elevation of body temperature (BT) after treatment, compared to the peak BT before treatment. C/I, casirivimab/imdevimab; R/D, remdesivir/dexamethasone. **, *p* < 0.005 vs. R/D.

**Table 2:** Clinical feature of patients treated by casirivimab/imdevimab in the late phase of SARS-CoV-2 infection.

Patient	Age	Sex	Underlying Disease	C/I Initiated Timing (Clinical Day)	Deterioration of Fever after C/I (Elevated Temperature) ^a^	Required Oxygen Therapy after C/I (Treated Days)	Length of Hospital Stay after Treatment (Days)
A	50	F	None	10	No	Yes (3)	6
B	55	M	None	6	Yes (≥1.0 °C)	Yes (4)	6
C	41	M	None	7	No	Yes (3)	5
D	46	M	None	9	Yes (<1.0 °C)	Yes (2)	4
E	44	M	None	7	Yes (<1.0 °C)	No	3
F	49	M	Allergic rhinitis	7	No	No	4

All patients in the late C/I group did not receive any treatment before C/I administration. ^a^, deterioration of fever was defined as at least ≥0.1 °C elevation of body temperature (BT) after treatment, compared to the highest BT before treatment. C/I, casirivimab/imdevimab.

## Data Availability

Not applicable.
